# TVAE-RNA: ensemble-based RNA secondary structure prediction via transformer variational autoencoders

**DOI:** 10.1093/bioinformatics/btaf527

**Published:** 2025-09-22

**Authors:** Xiyuan Mei, Hanbo Liu, Yuheng Zhu, Enshuang Zhao, Longyi Li, Hao Zhang

**Affiliations:** College of Computer Science and Technology, Jilin University, Changchun 130012, China; College of Computer Science and Technology, Jilin University, Changchun 130012, China; College of Computer Science and Technology, Jilin University, Changchun 130012, China; College of Computer Science and Technology, Jilin University, Changchun 130012, China; College of Computer Science and Technology, Jilin University, Changchun 130012, China; College of Computer Science and Technology, Jilin University, Changchun 130012, China

## Abstract

**Motivation:**

Accurate prediction of RNA secondary structure remains challenging due to the presence of pseudoknots, long-range dependencies, and limited labeled data.

**Results:**

We propose TVAE, a novel framework that integrates a Transformer encoder with a Variational Autoencoder (VAE). The Transformer captures global dependencies in the sequence, while the VAE models structural variability by learning a probabilistic latent space. Unlike deterministic models, TVAE generates diverse and biologically plausible secondary structures, enabling more comprehensive structure discovery. To obtain discrete predictions, we introduce GHA-Pairing, a fast and biologically constrained base-pairing algorithm. TVAE demonstrates strong generalization across different RNA families and achieves state-of-the-art performance on benchmark datasets, reaching an *F*1 score of 0.89 and 83% accuracy, surpassing existing methods by 10%. These results highlight the advantage of probabilistic modeling for RNA structure prediction and its potential to enhance biological insights.

**Availability and implementation:**

Code and pretrained models are available at https://github.com/mei-rna/TVAE-RNA. The released version of the dataset and models can also be accessed via DOI: 10.5281/zenodo.16946114.

## Graphical abstract

**Graphical Abstract:**
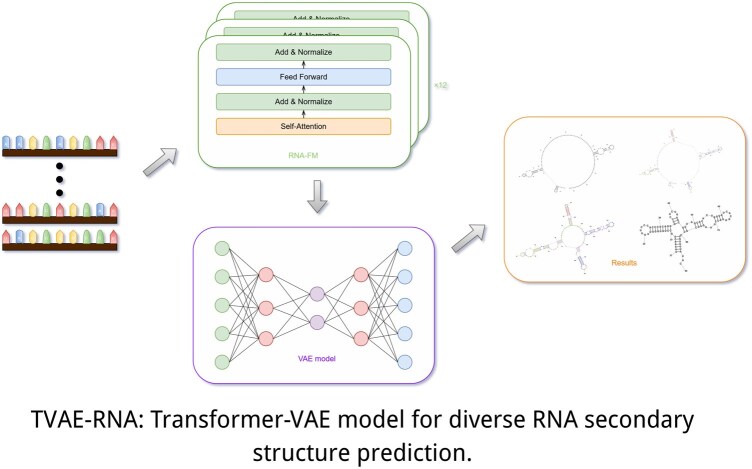

## 1 Introduction

RNA plays essential roles in gene regulation (Cao *et al.* 2024), translation (Holbrook 2005), and genome maintenance (Wan *et al.* 2011). Its biological functions are tightly linked to secondary structures (Higgs 2000), which govern interactions with proteins, ions, and small molecules (Svoboda and Cara 2006). Therefore, accurate prediction of RNA secondary structure is critical for understanding RNA biology (Butcher and Pyle 2011) and enabling therapeutic design (Gebauer *et al.* 2021).

Traditional methods—including dynamic programming algorithms (Nussinov and Jacobson 1980, Do *et al.* 2006), and chemical probing techniques (Hajdin *et al.* 2013, Ding *et al.* 2014, Tian and Das 2016)—thermodynamic models (Reuter and Mathews 2010, Lorenz *et al.* 2011)—face limitations in handling long sequences, pseudoknots, and data scarcity. Deep learning approaches, especially transformers (Zhao *et al.* 2021, Han *et al.* 2023) and VAEs (Bao *et al.* 2017, Burgess *et al.* 2018, Dai and Wipf 2019), have made significant progress by capturing long-range dependencies and modeling structural uncertainty. Hybrid models like KnotFold (Gong *et al.* 2024), SPOT-RNA (Singh *et al.* 2019), SPOT-RNA2 (Singh *et al.* 2021), RNAMoIP (Loyer and Reinharz 2024), and UFold (Fu *et al.* 2022) further improve performance through architectural integration or data fusion (Sato and Hamada 2023).

However, most existing models remain deterministic, predicting a single “optimal” structure, despite strong experimental evidence that RNA molecules adopt diverse structures under physiological conditions (Zhang *et al.* 2024). This limits their biological realism and therapeutic applicability.

To address these challenges, we propose TVAE, a Transformer-VAE hybrid that combines global sequence modeling with probabilistic latent representations. While our approach focuses on secondary structure diversity, we acknowledge that complete RNA structure ensembles involve tertiary structure dynamics, ligand-induced conformational changes, and environmental factors. Our work represents a first step toward ensemble-based prediction at the secondary structure level. TVAE generates diverse (Zhang *et al.* 2023), biologically plausible structures per RNA sequence (Cordero and Das 2015), capturing structural uncertainty and improving predictions for complex features like pseudoknots and non-canonical pairs.

We further introduce GHA-Pairing, a scalable post-processing algorithm for translating probabilistic outputs into discrete, interpretable secondary structures while preserving biological constraints.

In summary, TVAE advances RNA structure prediction by shifting from deterministic to diversity-aware modeling, offering a robust and biologically grounded framework for both basic and translational RNA research.

## 2 Materials and methods

### 2.1 Datasets and preprocessing

Our 150k pooled corpus includes sequences that overlap with the standard RNA-Strand and Rfam benchmark sets. To avoid any leakage, we treated these benchmarks as held-out evaluation resources: entries belonging to the standard RNA-Strand test set and to Rfam families designated for benchmarking were excluded from training/validation splits of BRNdata. We additionally report performance on these held-out benchmarks to verify robustness. Following common practice for reducing homologous bias in RNA benchmarks, we applied CD-HIT-EST at 80% identity to balance the removal of near-duplicate sequences with retention of family-level diversity. We will provide detailed filtering statistics (counts retained/removed by step; length distributions before/after) in Tables 1 and 2, available as supplementary data at *Bioinformatics* online, to justify the ∼87% reduction.

**Table 1.** TVAE compares *F*1 score, precision, and recall across nine methods.

Model	Precision	Recall	*F*1 score
**TVAE**	**0.828**	**0.8928**	**0.8592**
UFold	0.5681	0.6687	0.5953
SPOT-RNA	0.5417	0.5987	0.5778
RNAstructure	0.638	0.7487	0.6723
MXFold	0.5829	0.6434	0.6125
MXFold2	0.6101	0.7096	0.6433
KnotFold	0.7164	0.8327	0.7677
E2EFold	0.2697	0.2574	0.2531
RNAFold	0.5324	0.6487	0.5683

Bold values indicate the metrics of our model and represent the highest performance for each metric.

In this study, RNA sequences were collected from three public databases: bpRNA-1m (Danaee *et al.* 2018), Rfam (Griffiths-Jones *et al.* 2003), and lncRNAdb (Amaral *et al.* 2011), totaling over 150 000 entries across 4000+ RNA families. To improve model generalization, sequences were aggregated and processed via a standardized pipeline. Low-quality entries were removed, and FASTA files were reformatted uniformly. Redundancy was reduced using CD-HIT-EST (Li and Godzik 2006) (≥80% identity), and homologous sequences were filtered using BLAST-N (Altschul *et al.* 1997) (*E*-value ≤10). After deduplication, platform consistency analysis showed no significant batch effects, allowing for simple integration. From the cleaned data, 30% were sampled to construct BRNdata, a refined dataset with 18 328 unique RNA sequences.

### 2.2 RNA-FM embedding model

We employed RNA-FM (Chen *et al.* 2022), a 12-layer Transformer encoder pretrained with masked nucleotide prediction (15% masking rate), to extract RNA sequence embeddings. The model architecture (640-dimensional feedforward networks, 20-head self-attention) generates contextualized embeddings (*L* × 640 matrix) that simultaneously encode positional, structural, and functional features at nucleotide resolution.

### 2.3 Transformer-based variational autoencoder model

To capture long-range dependencies and structural variability in RNA, we propose TVAE, a Transformer-based Variational Autoencoder. It processes RNA-FM embeddings through a shared Transformer encoder–decoder architecture, augmented with dynamic attention spans and relative position encoding. A latent variable is sampled via the reparameterization trick and used to reconstruct base-pairing matrices. The overall workflow is outlined in Algorithm 1.

Algorithm 1: Transformer-VAE Workflow with Dynamic Attention and Relative Positional Encoding Input: RNA-FM embedding E ∈ ℝ^{L×640}

Output:Predicted base-pairing probability matrix P ∈ RL×L

1. Embedding Projection

   H ← Linear(E) → Activation → LayerNorm

2. Transformer Encoder with Dynamic Attention

   For each Transformer encoder layer:

    a. For each position i:

     - Compute dynamic attention span:

     span ← min(S_max, 1 + growth_rate * i)

     - Define attention mask:

     mask[i] ← [j for j in range(i - span, i + span +

1)]

   b. For each (i, j) in attention:

    # Relative position bias (proposed)

    - Compute attention score with relative bias:

    AttnScore[i][j] ← Q[i] • K[j] + RelBias[i - j]

   c. Apply Multi-head Attention + Residual + FFN +LayerNorm

3. Latent Variable Sampling (VAE Module)

   - Compute mean and variance:

     Z_mean, Z_logvar ← Linear(H)

   - Reparameterization:

     Sample Z ← Z_mean + ε * exp(0.5 * Z_logvar)

4. Transformer Decoder

   For each Transformer decoder layer:

     Apply Multi-head Attention + Residual + FFN +

LayerNorm

5. Output Layer

   - Compute base-pairing probabilities:

     Output base-pairing matrix ← Sigmoid(Linear(Z))

**Algorithm 1**. The workflow integrates two novel mechanisms to enhance RNA structure modeling:

1. Dynamic Attention Span, which adapts attention windows based on nucleotide position to balance locality and efficiency in long sequences;
2. Relative Positional Encoding, which adds learnable distance-aware bias to attention scores, improving the modeling of RNA folding constraints.

These innovations allow the model to better capture long-range and context-specific interactions critical to secondary structure prediction.

**Figure 1.**
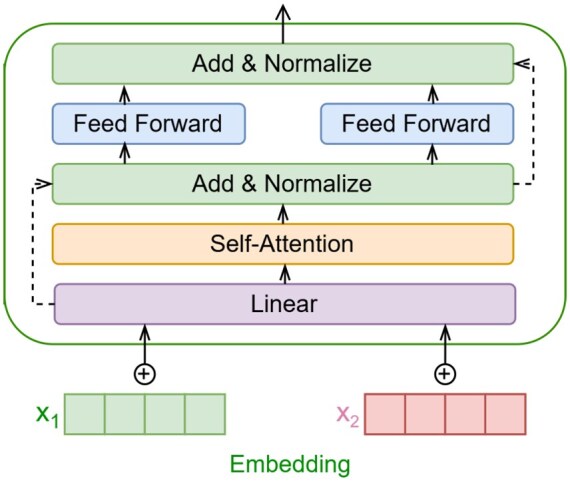
The Transformer architecture includes a linear layer, multi-head self-attention, feedforward network, and normalization.

An overview of the Transformer-based architecture is illustrated in Fig. 1, which shows the full data flow of our proposed TVAE framework. The model takes RNA-FM embeddings as input and applies a shared Transformer encoder–decoder backbone, augmented with a dynamic attention span mechanism and relative positional encoding. The encoder generates contextual representations and estimates the latent distribution parameters (mean and variance), from which a latent variable is sampled. The decoder reconstructs a base-pairing matrix from the sampled latent variable. This architecture enables diverse and biologically plausible RNA secondary structure predictions.

**Figure 2.**
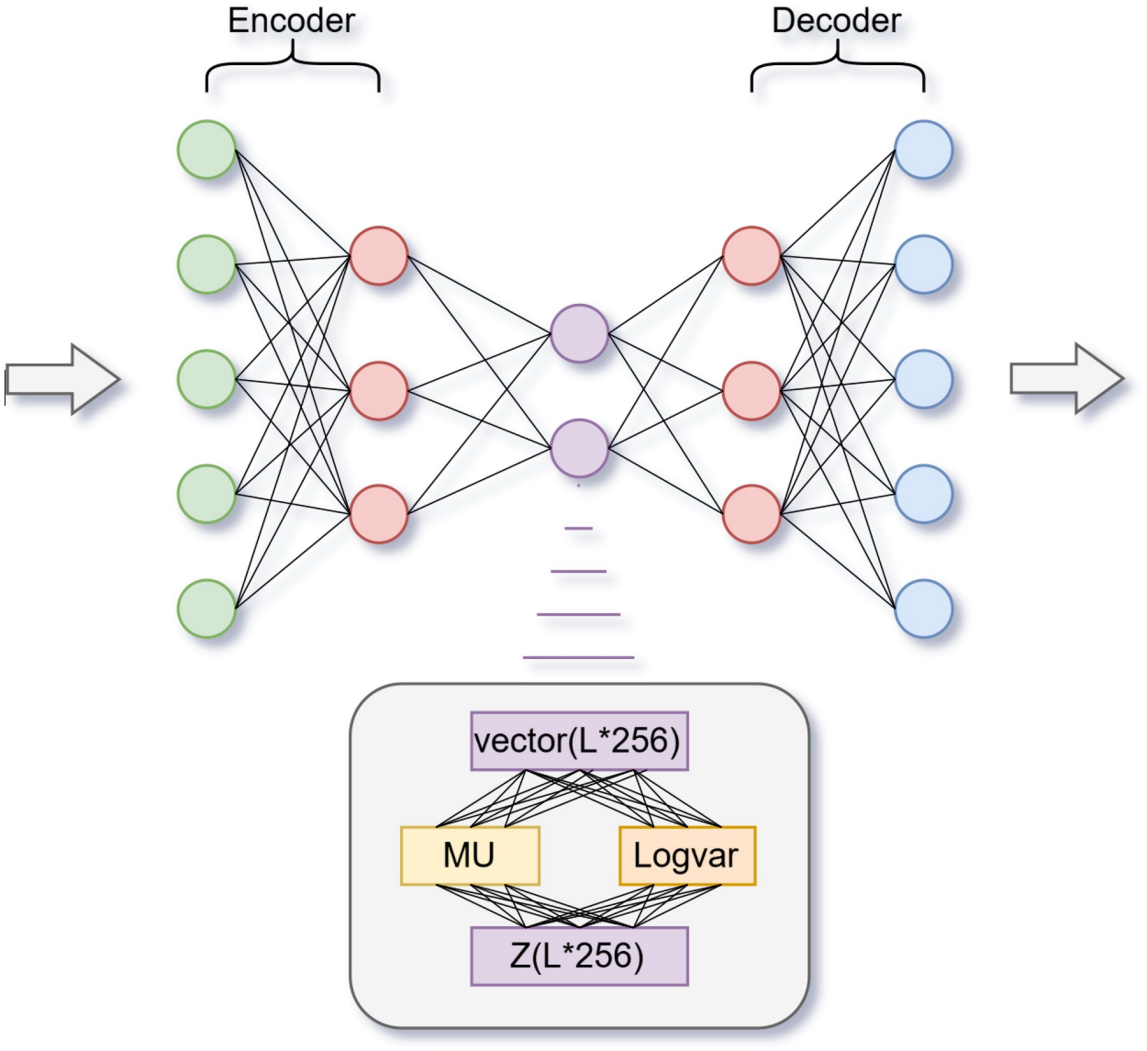
The VAE uses Transformer-based encoder and decoder. The encoder generates latent variable *Z*, which the decoder uses to predict RNA secondary structure.

Beyond the Transformer architecture, TVAE introduces a variational inference component to model structural diversity. As illustrated in Fig. 2, the encoder outputs are mapped to the mean and variance of a latent variable distribution, from which a latent vector is sampled using the reparameterization trick. This sampled latent representation captures the uncertainty and variability of RNA folding and is subsequently decoded to reconstruct the base-pairing probability matrix. The variational component enables the generation of diverse yet biologically plausible RNA secondary structures.

In summary, the TVAE framework integrates a Transformer-based encoder–decoder backbone with variational inference, enabling the model to capture both global sequence dependencies and structural uncertainty. The incorporation of dynamic attention span and relative positional encoding enhances modeling of RNA-specific structural constraints, while the probabilistic latent space allows for diverse and biologically meaningful predictions. This architectural design lays the foundation for accurate, robust, and diversity-aware RNA secondary structure modeling.

### 2.4 Loss function

The model is trained end-to-end using a loss function combining reconstruction loss and KL divergence to capture RNA’s probabilistic structure.

The KL divergence is used to measure the distance between the latent variable distribution $q(z|x)=N(\mu ,\sigma^{2})$ generated by the encoder and the standard normal distribution p(z)=N(0, I).  The expression is as follows:


(1) $$L_{\mathrm{KL}}=\frac{1}{2}\sum_{i=1}^{L}\left(1+\;\log\;\sigma_{i}^{2}-\mu_{i}^{2}-\sigma_{i}^{2}\right)$$

We apply masking to exclude padding regions and ensure numerical stability to prevent NaNs from log or float errors. The KL loss is batch-averaged and negated during training.

The reconstruction loss $L_{\mathrm{recon}}$ is calculated using the mean absolute error (MAE) between the reconstructed output and the target matrix, formulated as follows:


(2) $$L_{\mathrm{recon}}=\sum_{i=1}^{N}|y_{\mathrm{pred}}^{\left(i\right)}-y_{\mathrm{true}}^{\left(i\right)}|$$

where N denotes the total number of elements in the matrix, $y_{\mathrm{pred}}^{\left(i\right)}\;\mathrm{and}\;y_{\mathrm{true}}^{\left(i\right)}$ represent the values at the *i*th position in the reconstructed output and target matrix, respectively.

The final loss function of the VAE model is obtained by summing the reconstruction loss and the regularization loss, and then multiplying by a scaling coefficient:


(3) $$L=\alpha L_{\mathrm{recon}}+\beta L_{KL}$$

The weights *α* and *β* in Equation (3) were empirically determined via grid search on the validation set. To assess robustness, we conducted sensitivity analysis by varying *α* and *β* within the range [0, 1.0]. Figure 1, available as supplementary data at *Bioinformatics* online, shows that model performance is stable across this range, with *α* = 0.9 and *β* = 0.1 providing the best balance between reconstruction accuracy and KL regularization.

**Figure 3.**
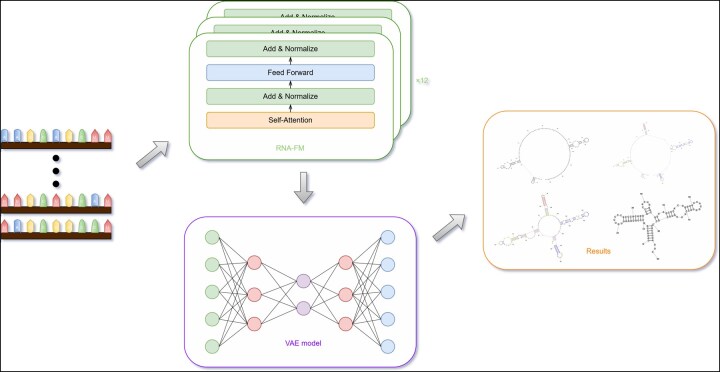
The full training pipeline. Starting from RNA sequence input, the framework uses the RNA-FM and VAE models to predict RNA secondary structure.

The framework integrates RNA-FM embeddings with a Transformer-based VAE. The encoder maps sequences to a latent space, and the decoder reconstructs RNA structures from sampled variables. Both are trained jointly to minimize reconstruction and KL divergence losses, enabling diverse and meaningful structural predictions (see Fig. 3).

Training the VAE-based model significantly enhances its adaptability to diverse RNA sequence distributions. Experimental results demonstrate that our method consistently outperforms current state-of-the-art approaches, particularly on RNA families not seen during training. This improved generalization is reflected in higher prediction accuracy across various RNA types, underscoring the model’s robustness and broad applicability in RNA secondary structure prediction.

### 2.5 GHA-Pairing: Greedy Hungarian approximation for RNA pairing

To efficiently convert predicted base-pairing probability matrices into discrete RNA secondary structures, we propose GHA-Pairing, a fast greedy approximation of the Hungarian algorithm (Mills-Tettey *et al.* 2007). This method preserves high-quality pairings while substantially reducing computational cost.

Given a predicted foreground pairing probability matrix $P^{\mathrm{fg}}\in R^{L\times L}$ and a background pairing probability matrix $P^{\mathrm{bg}}\in R^{L\times L}$, GHA-Pairing constructs a score matrix $S\in R^{L\times L}$ by computing the log-odds contrast between foreground and background as follows:


(4) $$S_{i,j}=\log\left(\frac{P_{i,j}^{fg}}{P_{i,j}^{bg}}\right)+\log(\frac{1-P_{i,j}^{bg}}{1-P_{i,j}^{fg}})-\varphi$$

where φ is a penalty term used to discourage weak or spurious pairings. This scoring function encourages pairings that are consistently favored by the model, while penalizing uncertain or conflicting evidence.

The algorithm then sorts all valid candidate pairs (i, j) that satisfy base-pairing constraints in descending order of $S_{i,j}$. Starting from the highest-scoring pair, the algorithm greedily assigns the pair if neither base has been paired before. This process continues until no further valid assignments can be made.

The GHA-Pairing algorithm is highly efficient and scalable, with a time complexity of approximately$\;O(n^{2}\log n)$, significantly lower than the classical Hungarian algorithm’s $O(n^{3})$. Despite its simplified greedy approach, GHA-Pairing achieves accuracy comparable to or surpassing more complex optimization methods. Thus, it offers a fast, accurate, and stable solution for converting predicted pairing probabilities into precise RNA secondary structures.

## 3 Results

### 3.1 Dataset and preprocessing results

#### 3.1.1 Dataset statistics

For benchmark evaluation, we reserve the standard RNA-Strand test sequences and a disjoint set of Rfam families strictly excluded from training of BRNdata. This design ensures representativeness while preventing train–test leakage. Results are summarized in the main text, Table 3, available as supplementary data at *Bioinformatics* online.

We constructed BRNdata, a benchmark dataset for RNA secondary structure prediction, by integrating sequences from bpRNA-1m (Danaee *et al.* 2018), Rfam (Griffiths-Jones *et al.* 2003), and lncRNAdb (Amaral *et al.* 2011). BRNdata contains 18 328 sequences mostly between 50 and 512 nucleotides (average ∼200). The dataset is split into training (70%), validation (20%), and test (10%) sets. After filtering low-quality and redundant sequences, the final dataset is balanced and representative.

#### 3.1.2 Data preprocessing and redundancy removal

We add step-wise filtering statistics (deduplication, CD-HIT-EST @80%, BLAST-N filtering) to quantify contributions to the final size. We provided sequence length distributions before and after deduplication in Figs 2 and 3, available as supplementary data at *Bioinformatics* online, supporting the representativeness of BRNdata.

To ensure robust model generalization, we implemented rigorous redundancy removal procedures. Specifically, we applied CD-HIT-EST and BLAST-N with an 80% sequence similarity threshold to eliminate potentially homologous sequences. This preprocessing step reduced the dataset size by 70% while significantly enhancing sequence diversity and minimizing overfitting risks.

### 3.2 Model performance

#### 3.2.1 Prediction accuracy evaluation

We conducted comprehensive performance evaluation using three widely accepted metrics in RNA structure prediction: precision, recall, and *F*1 score. These metrics provide complementary perspectives on prediction quality, defined as:


(5) $$\mathrm{Prec}=\frac{\mathrm{TP}}{\mathrm{TP}+\mathrm{FP}}$$

(6) $$\mathrm{Recall}=\frac{\mathrm{TP}}{\mathrm{TP}+\mathrm{FN}}$$

(7) $$F1=2\times \frac{\mathrm{Recall}\times \mathrm{Prec}}{\mathrm{Recall}+\mathrm{Prec}}$$

TP, FP, and FN denote true positives (correctly predicted pairs), false positives (incorrect predictions), and false negatives (missed reference pairs), respectively.

To assess the effectiveness of our proposed TVAE model, we conducted a comprehensive comparison against eight representative baseline methods—E2Efold (Shen *et al.* 2022), Knotfold (Gong *et al.* 2024), MXfold2 (Sato *et al.* 2021), MXfold (Akiyama *et al.* 2018), RNAfold (Lorenz *et al.* 2011), RNAstructure (Reuter and Mathews 2010), SPOT-RNA (Singh *et al.* 2019), and UFold (Fu *et al.* 2022)—on an identical test dataset. These methods span a range of strategies, including end-to-end deep learning, pseudoknot modeling, thermodynamic integration, and multimodal feature fusion.

**Figure 4.**
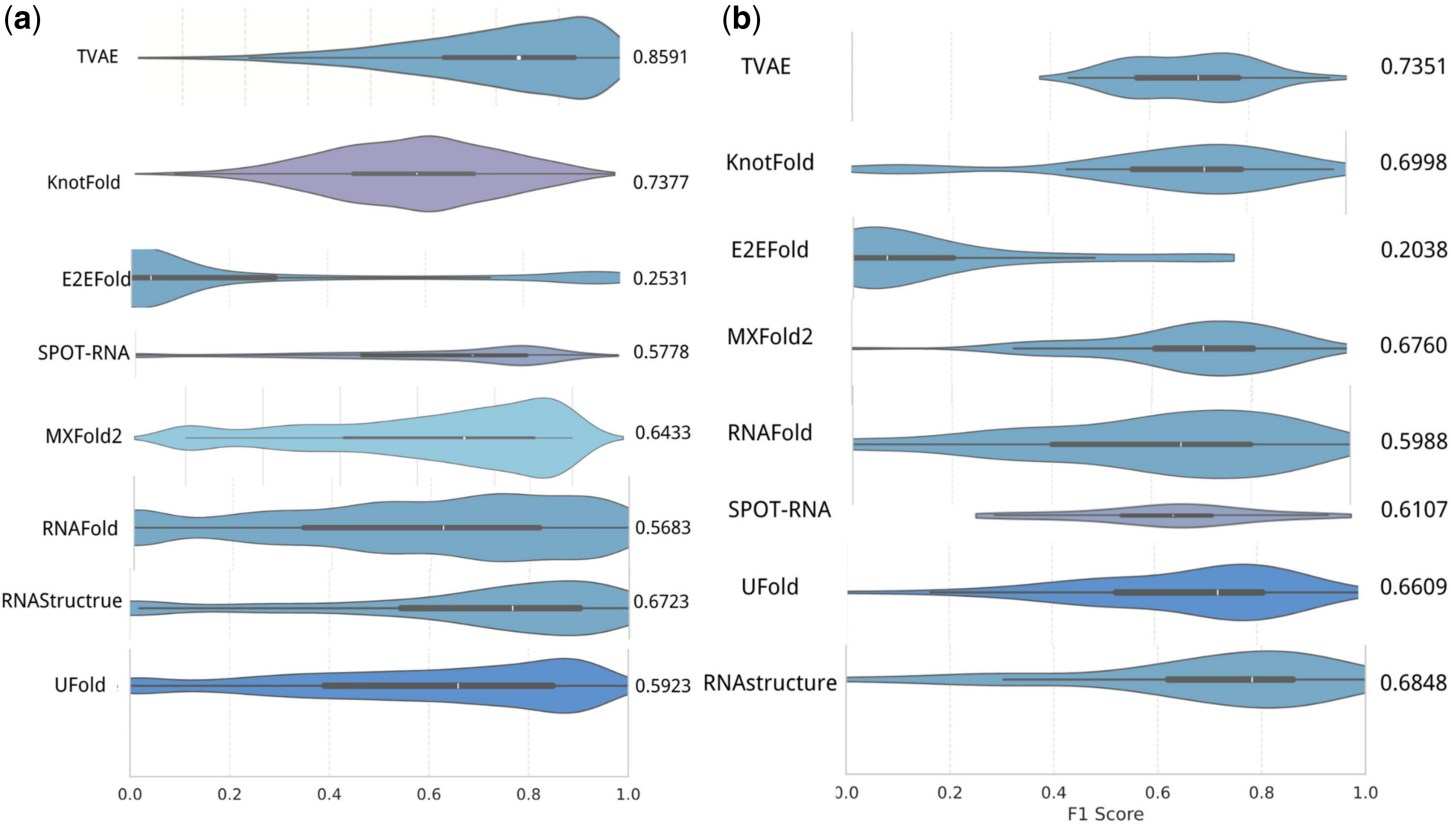
Violin plots comparing *F*1 score distributions of TVAE and baseline methods. (a) Results on the standard test dataset. (b) Results on the independent PDB dataset, which contains RNA sequences not seen during training. TVAE consistently achieves higher and more stable scores across both datasets, demonstrating strong predictive accuracy and generalization.

TVAE consistently outperforms baseline models across multiple metrics. As shown in Table 1, it achieves an *F*1 score of 0.8592, precision of 0.8280, and recall of 0.8928 on the test set. To assess generalization, we further evaluated the model on the independent PDB dataset containing sequences unseen during training. The *F*1 score distributions shown in Fig. 4 demonstrate that TVAE not only yields high accuracy but also maintains stable performance across diverse RNA families on both datasets.

Table 1 compares *F*1 score, precision, and recall across nine methods, with TVAE achieving the best performance on all metrics.

**Figure 5.**
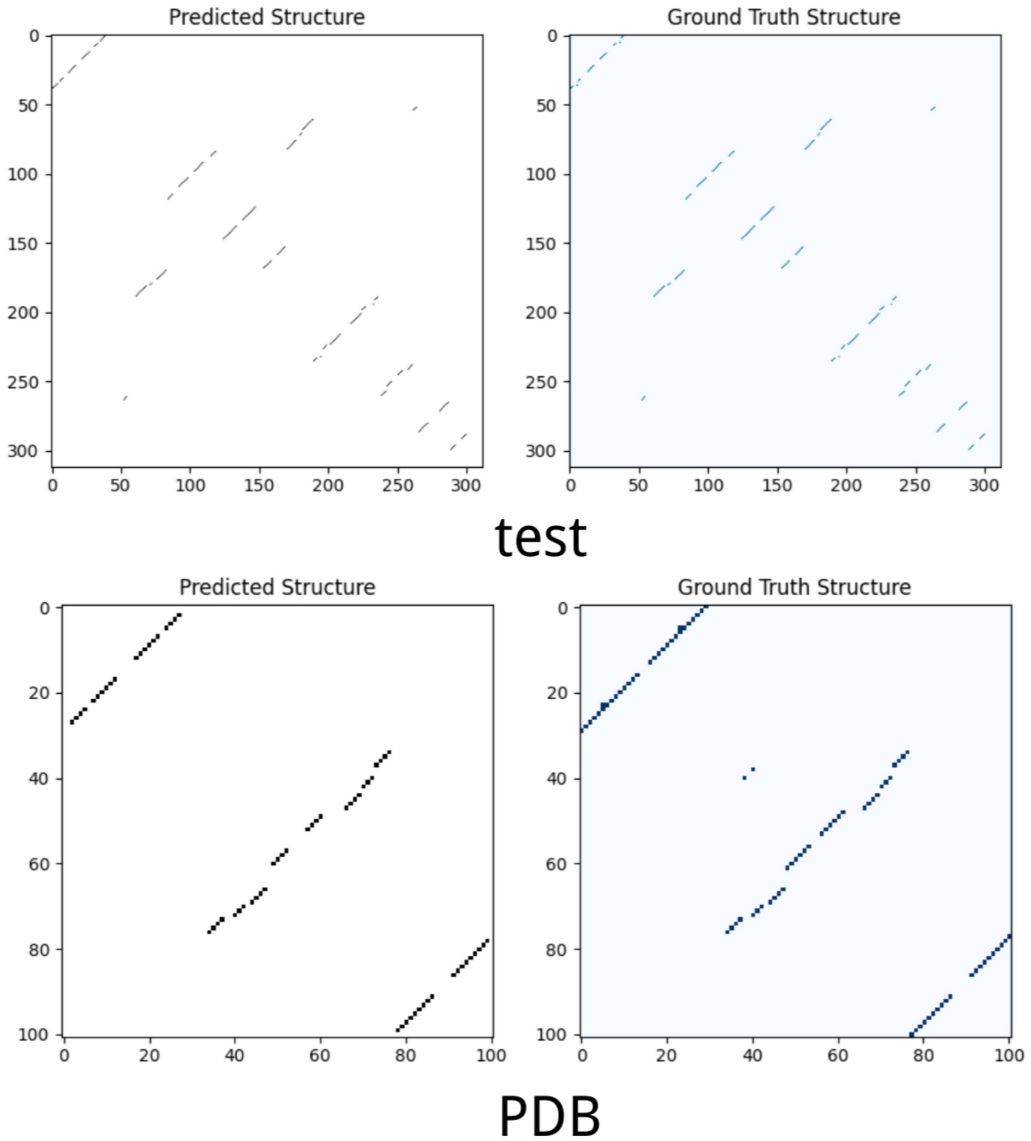
Visual comparison between predicted and ground truth pairing matrices.

To illustrate structural prediction performance, we visualized the predicted and reference base-pairing matrices of two representative sequences from the PDB and test sets (Fig. 5). The results highlight the accuracy and robustness of our method.

**Figure 6.**
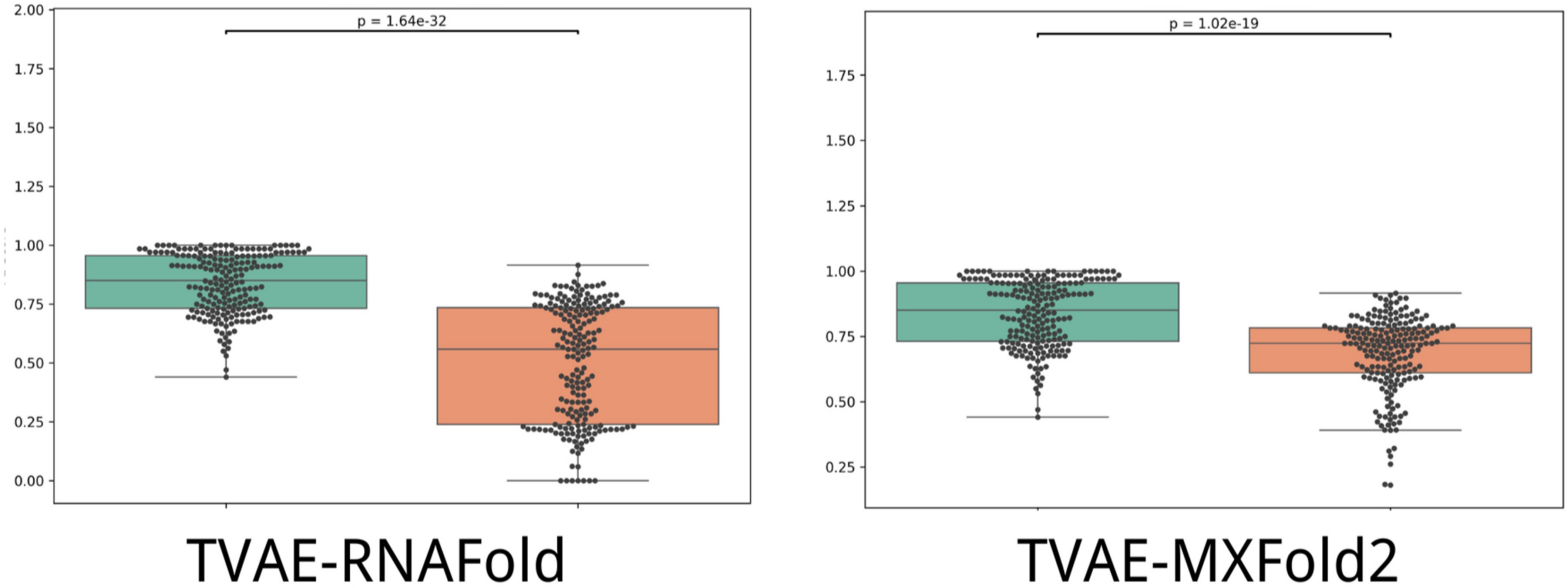
Wilcoxon signed-rank test results between TVAE, MXFold2, and RNAfold on the same test set. TVAE significantly outperforms the baselines on most samples (*P* < .01).

To further assess TVAE’s performance on the test set, we conducted Wilcoxon signed-rank tests (Taheri and Hesamian 2013) against MXFold2 and RNAfold using *F*1 scores. Results show that TVAE significantly outperforms both baselines on the test set (*P* < .01), with consistently higher and more stable *F*1 distributions (Fig. 6), demonstrating stronger structural modeling and generalization capabilities.

In summary, we systematically compared TVAE with state-of-the-art baselines (e.g. SPOT-RNA, RNAfold) across multiple RNA datasets. Violin plots and Wilcoxon signed-rank tests (*P* < .01) demonstrate that TVAE consistently achieves higher accuracy and stability. These advantages stem from the integration of Transformer and VAE architectures, enabling better modeling of latent RNA structural patterns. TVAE thus offers a robust and promising solution for RNA secondary structure prediction.

In addition to Wilcoxon signed-rank tests, we report 95% confidence intervals and effect sizes (Cohen’s *d*) for pairwise comparisons, with multiple testing corrections applied using the Benjamini–Hochberg procedure to control the false discovery rate. The results of these statistical analyses are provided in Figs 4 and 5 and Tables 4 and 5, available as supplementary data at *Bioinformatics* online, which clearly demonstrate that TVAE achieves consistently higher *F*1 scores compared to RNAFold and MXFold2.

#### 3.2.2 Prediction visualization

**Figure 7.**
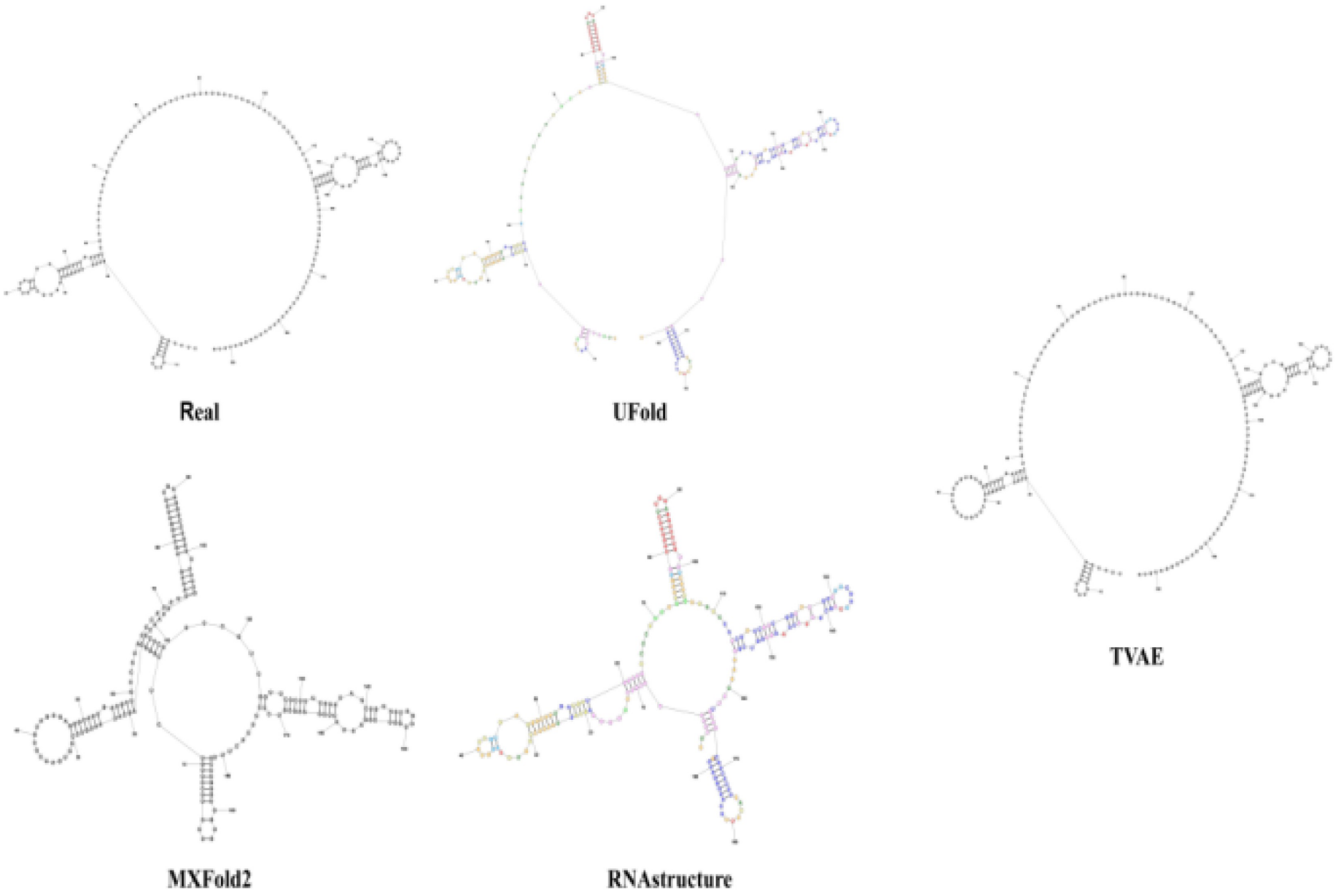
The picture shows the visual comparison between the RNA secondary structures predicted by the four methods and the ground truth.

To demonstrate TVAE’s superior modeling, we selected representative RNA sequences and compared predicted secondary structures (Fig. 7). Using standard arc notation from the RNAstructure web server (Reuter and Mathews 2010), the diagrams show TVAE’s improved accuracy over UFold, MXFold2, and RNAstructure in capturing complex base-pairing. Figure 7 highlights TVAE’s ability to model complex RNA structures.

**Figure 8.**
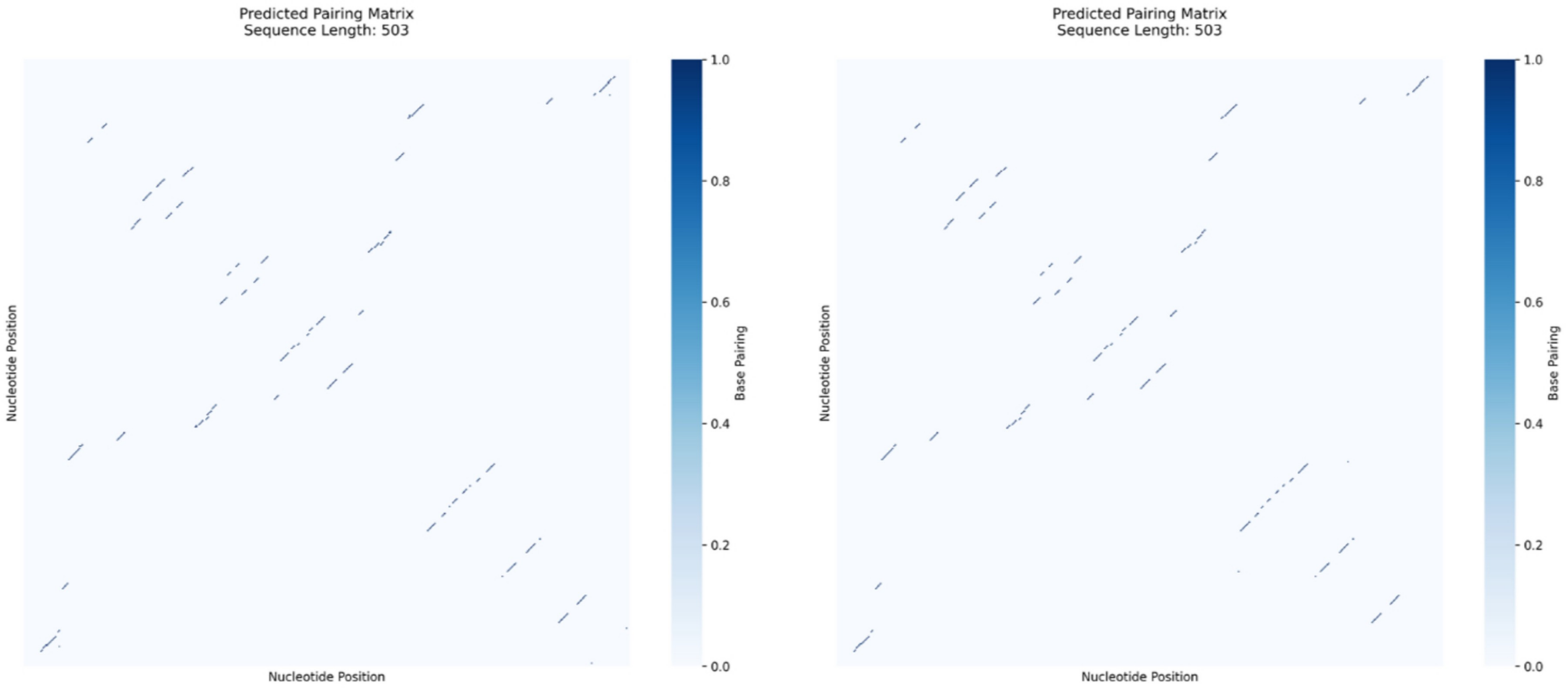
Two predicted secondary structures for the same RNA sequence by TVAE are mostly similar with subtle differences, showing the model’s ability to generate diverse yet reasonable outputs.

To highlight TVAE’s ability to generate multiple distinct structures for one RNA sequence, we show in Fig. 8 different base-pairing matrices from multiple latent space samplings. This demonstrates TVAE’s capacity to model structural heterogeneity with diverse, biologically plausible conformations.

### 3.3 Generative diversity and biological plausibility

A distinguishing feature of TVAE is its ability to generate diverse, biologically meaningful RNA secondary structures for each input sequence by probabilistically sampling from the learned latent space. This diversity directly addresses a key limitation of deterministic models, which produce a single “optimal” prediction and fail to capture the range of structural possibilities observed in experimental studies such as SHAPE-MaP (Weeks 2010) and mutate-and-map assays (Rouskin *et al.* 2014).

Importantly, such structural diversity is biologically functional, not an artifact of modeling. Numerous studies have shown that variations in RNA folding play essential roles in regulatory processes like riboswitch activation, translational attenuation, and splice site accessibility (Breaker 2012). For instance, the HIV-1 5′ UTR dynamically shifts its secondary structure to regulate translation versus packaging (Watts *et al.* 2009), and RNA viruses often exploit structural variability to evade immune detection (Dethoff *et al.* 2012).

By embracing diversity in structural prediction, TVAE enables more comprehensive identification of functionally relevant patterns. In the context of siRNA and antisense oligonucleotide (ASO) design, e.g. consistently exposed binding regions across diverse structural predictions can increase targeting efficiency and reduce off-target effects (Kamola *et al.* 2015). Similarly, in RNA vaccine design, the ability to identify stable and accessible epitopes across multiple predicted structures enhances immunogenicity and robustness (Damase *et al.* 2021).

In summary, TVAE extends beyond accuracy metrics by offering a diversity-aware framework that supports functional interpretation and therapeutic innovation. Its probabilistic structure generation enables exploration of alternative but plausible RNA foldings, paving the way for RNA-based precision medicine strategies sensitive to genetic variation and environmental context.

To quantify structural diversity, we implemented two complementary metrics based on sampled conformations. First, we estimated base-pairing probabilities ${\;P}_{ij}$ from K sampled structures, from which we derived per-position entropy $H_{i}$ and reported the global entropy$\;H_{\mathrm{global}}$. Second, we calculated the Pairwise Structural Diversity Index (PSDI), defined as the mean base–pair distance between all pairs of sampled structures. These two measures together capture both local uncertainty and global conformational variability. As summarized in Table 6, available as supplementary data at *Bioinformatics* online, our results demonstrate that TVAE generates conformational ensembles with substantial entropy and non-trivial PSDI values, highlighting its ability to produce structurally diverse RNA secondary structures.

In terms of biological validation, we emphasize that entropy and PSDI are widely recognized metrics for characterizing RNA conformational heterogeneity. Elevated entropy values reflect local flexibility at individual nucleotide positions, while larger PSDI values capture substantial structural differences across sampled ensembles. Together, these measures align with established principles of RNA structural dynamics and support the biological plausibility of the diversity generated by TVAE. Moreover, SHAPE-MaP experimental ensembles serve as an important benchmark for assessing conformational heterogeneity. Although direct integration of SHAPE-MaP data is beyond the scope of the present study, our framework is readily extensible to incorporate such datasets in future work, which will further enhance the biological relevance of the predicted structural diversity.

### 3.4 Ablation analysis

We conducted ablation studies to evaluate the impact of key architectural choices, including input representations (RNA-FM versus one-hot) and VAE regularization (with versus without KL divergence). All models were trained under identical conditions for fair comparison. Notably, removing KL divergence reduces the model to a deterministic Transformer, enabling direct assessment of diversity and generative capacity.

**Figure 9.**
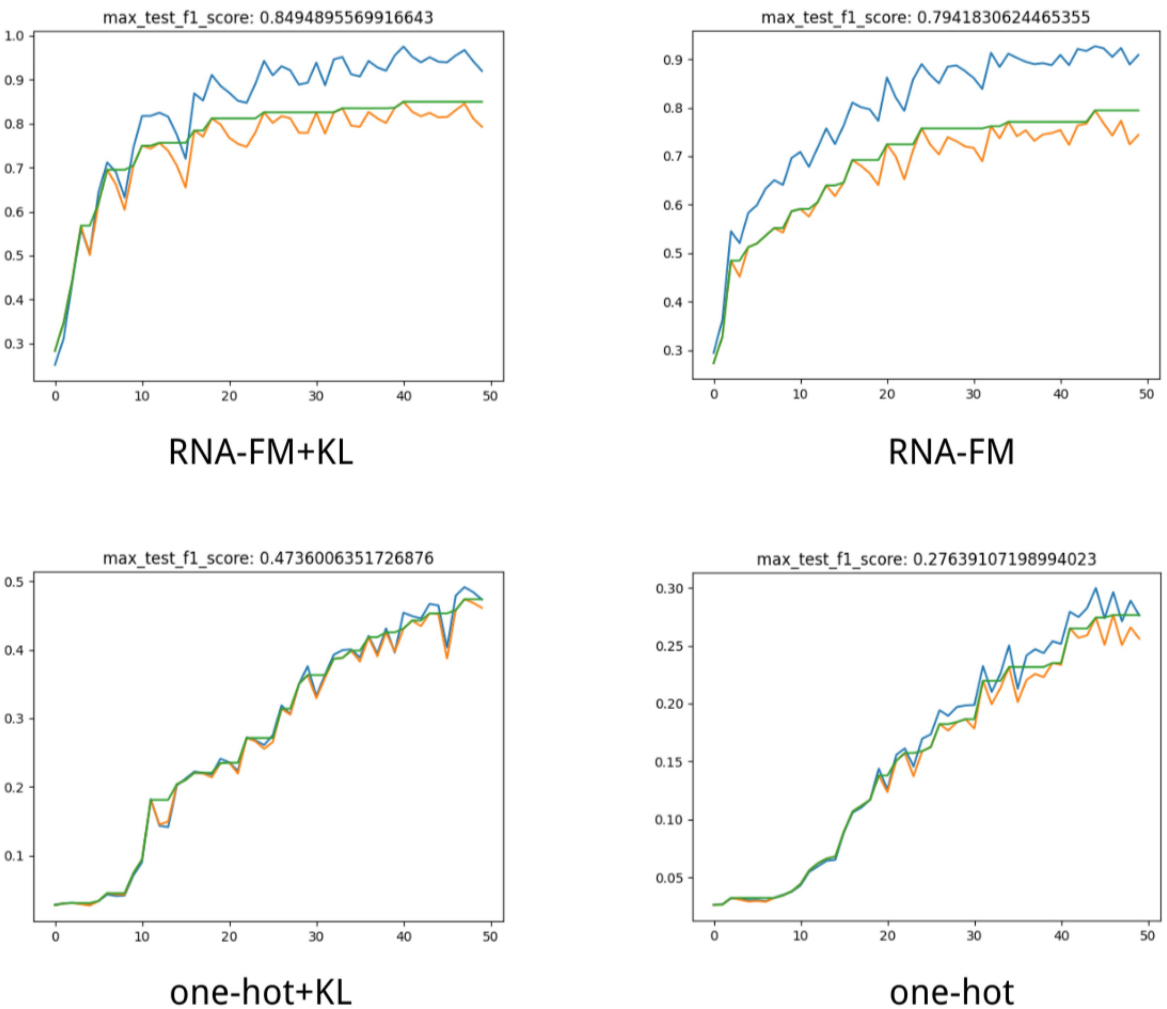
*F*1 score progression during training under four ablation settings.

Results show that RNA-FM embeddings consistently lead to better performance, highlighting the value of pretrained features. Incorporating KL divergence further improves generalization, especially with one-hot inputs. To illustrate training dynamics, we plotted *F*1 score trajectories (Fig. 9), showing that KL-regularized models converge more smoothly and reach higher final accuracy, demonstrating the benefit of variational modeling.

In addition to the ablation experiments, we further analysed the impact of different *α* and *β* weight settings. The results confirm that the ablation conclusions remain valid across a broad range of weight values, demonstrating that the benefits of KL divergence are not sensitive to the precise choice of *α* and *β*.

### 3.5 Hyperparameter settings and training details

To balance performance and model size, we set the embedding dimension to 256 and used four Transformer encoder layers with eight attention heads, totaling ∼3 million parameters. This configuration was chosen as a tradeoff between accuracy and efficiency. While larger models with more layers and attention heads provided marginal improvements, the computational cost increased substantially. Therefore, we adopted the reported configuration as the optimal balance for practical training and evaluation.

TVAE was trained on two NVIDIA RTX 3080 GPUs (16 GB) with a batch size of 4 for 400 000 steps. We used the AdamW optimizer with a 0.0001 learning rate, *β*_1_ = 0.9, *β*_2_ = 0.999, and L2 weight decay of 0.01.

### 3.6 Computation optimization and model compression

#### 3.6.1 Model compression results

To evaluate computational scalability, we compared the wall-clock time of TVAE with baseline methods across different sequence lengths (128–1024 nt). Results in Table 7, available as supplementary data at *Bioinformatics* online, demonstrate that TVAE achieves very high efficiency, requiring only a fraction of a second to predict a single sequence, which is substantially faster than most mainstream methods. Moreover, TVAE scales approximately linearly with sequence length and remains feasible up to 1024 nt on a single 16 GB GPU. For sequences longer than 512 nt, we employed a segmented prediction strategy that partitions the input into overlapping windows, followed by structure stitching to ensure global consistency (Fan *et al.* 2024). Furthermore, we systematically investigated the trade-off between ensemble size and performance. As shown in Fig. 6, available as supplementary data at *Bioinformatics* online, generating 10–20 samples provides an optimal balance between structural diversity and predictive accuracy, while larger ensembles yield diminishing returns.

To enhance inference efficiency, we applied model quantization and pruning. These techniques reduced weight precision and removed redundant parameters, respectively, yielding a 30% reduction in model size and 40% lower FLOPs. The optimized model achieved up to 5× faster inference with less than 5% accuracy loss, maintaining competitive performance for practical deployment.

#### 3.6.2 Knowledge distillation for improved computational efficiency

To improve scalability, we used knowledge distillation to create a lightweight student model. It learns from ground truth and TVAE’s soft predictions, inheriting ensemble capabilities in compressed form. This yields a three-fold speedup while retaining 90% of original accuracy, enabling efficient deployment in resource-limited settings and demonstrating knowledge distillation’s practical value for RNA structure prediction.

## 4 Discussion

In this study, we proposed TVAE, a Transformer-based Variational Autoencoder designed to address key challenges in RNA secondary structure prediction, including long-range dependencies, pseudoknot modeling, and structural variability. By integrating a self-attention-based encoder–decoder backbone with probabilistic latent space modeling, TVAE captures both global contextual information and intrinsic structural uncertainty, enabling the generation of diverse, biologically plausible secondary structures.

Unlike traditional deterministic models that output a single “optimal” structure, TVAE leverages variational inference to learn a latent distribution, from which multiple structure candidates can be generated. This allows the model to identify regions of structural ambiguity, which may correspond to flexible, functionally important RNA segments. The ability to reflect such uncertainty is particularly valuable in cases where experimental data is sparse or conflicting, making TVAE a useful tool for guiding further biological investigation.

While our model shows strong predictive performance across multiple datasets, including unseen RNA families, there are several limitations. First, TVAE is limited to secondary structure prediction and does not incorporate tertiary interactions or account for structural changes induced by proteins, ligands, or environmental factors such as temperature, pH, and ionic concentration. Second, the model does not capture RNA kinetic folding pathways, which may influence structural equilibria in vivo. Future extensions could integrate high-throughput structure probing data, folding kinetics, or 3D constraints to enhance the biological realism and scope of the framework.

Despite these limitations, this work represents a meaningful first step toward uncertainty-aware and diversity-enabled RNA structure prediction. The proposed TVAE model not only achieves high prediction performance but also provides valuable analytical capabilities for RNA biology and therapeutic applications. First, it can assist experimental design by highlighting structurally uncertain regions that merit further investigation. Second, it supports mutation prioritization by identifying sequence positions where changes are likely to induce structural shifts. Third, it enables confidence estimation by quantifying predictive variability, thereby improving the interpretability and reliability of structure predictions in downstream tasks. Together, these features make TVAE a practical and versatile tool for both fundamental research and translational studies in RNA science.

## Supplementary Material

btaf527_Supplementary_Data

## Data Availability

Code and pretrained models are available at https://github.com/mei-rna/TVAE-RNA. The released version of the dataset and models can also be accessed via DOI: 10.5281/zenodo.16946114.
