# Supplementary figures and images for: TVAE-RNA: ensemble-based RNA secondary structure prediction via transformer variational autoencoders
Source: Bioinformatics. 2025 Sep 22;41(11):btaf527. doi: 10.1093/bioinformatics/btaf527 (PMC12640237; doi:10.1093/bioinformatics/btaf527)

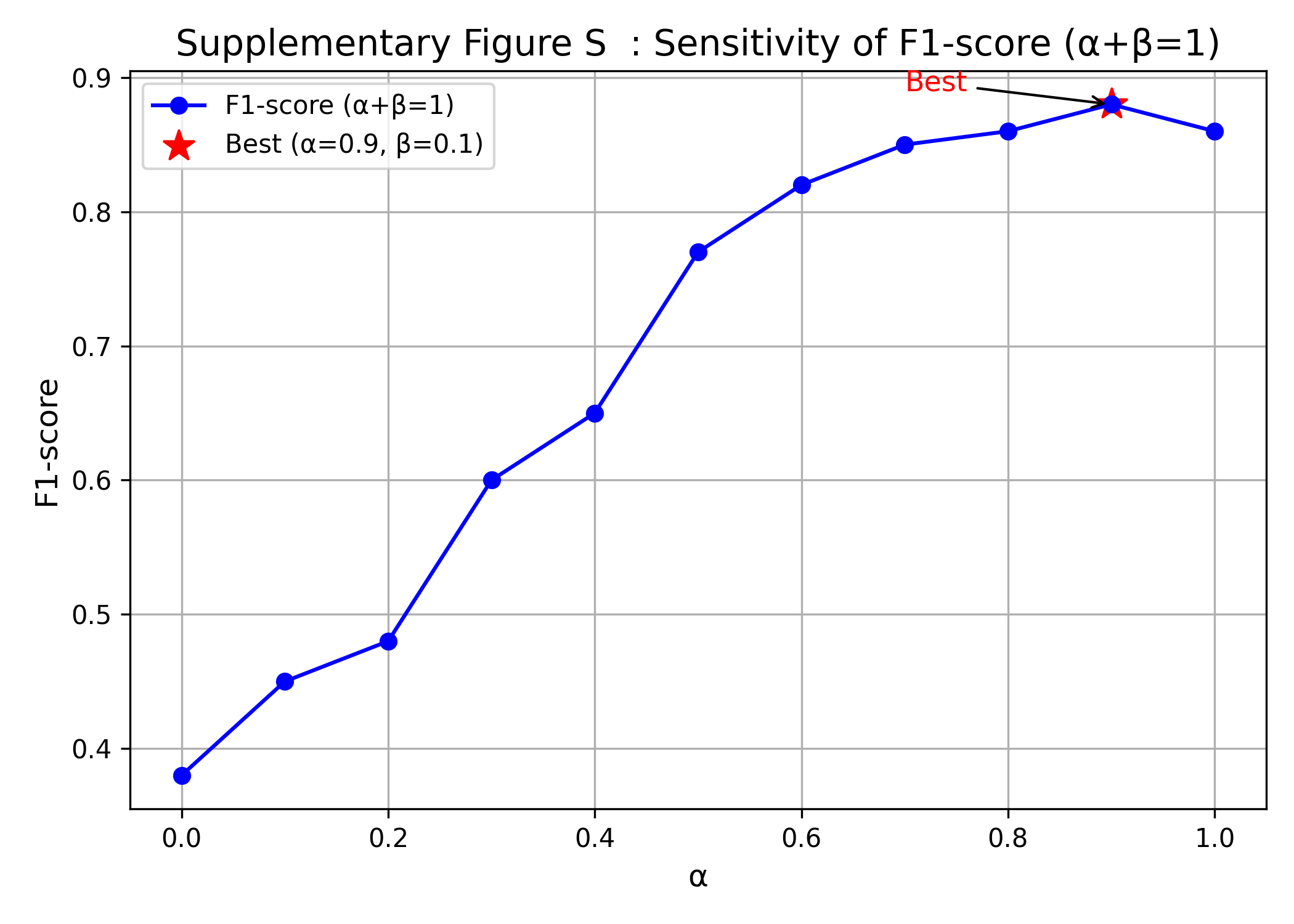

Supplement: btaf527_Supplementary_Data [file btaf527_supplementary_data.zip › Supplementary material/Supplementary Materials for Online Figures S1.bmp]

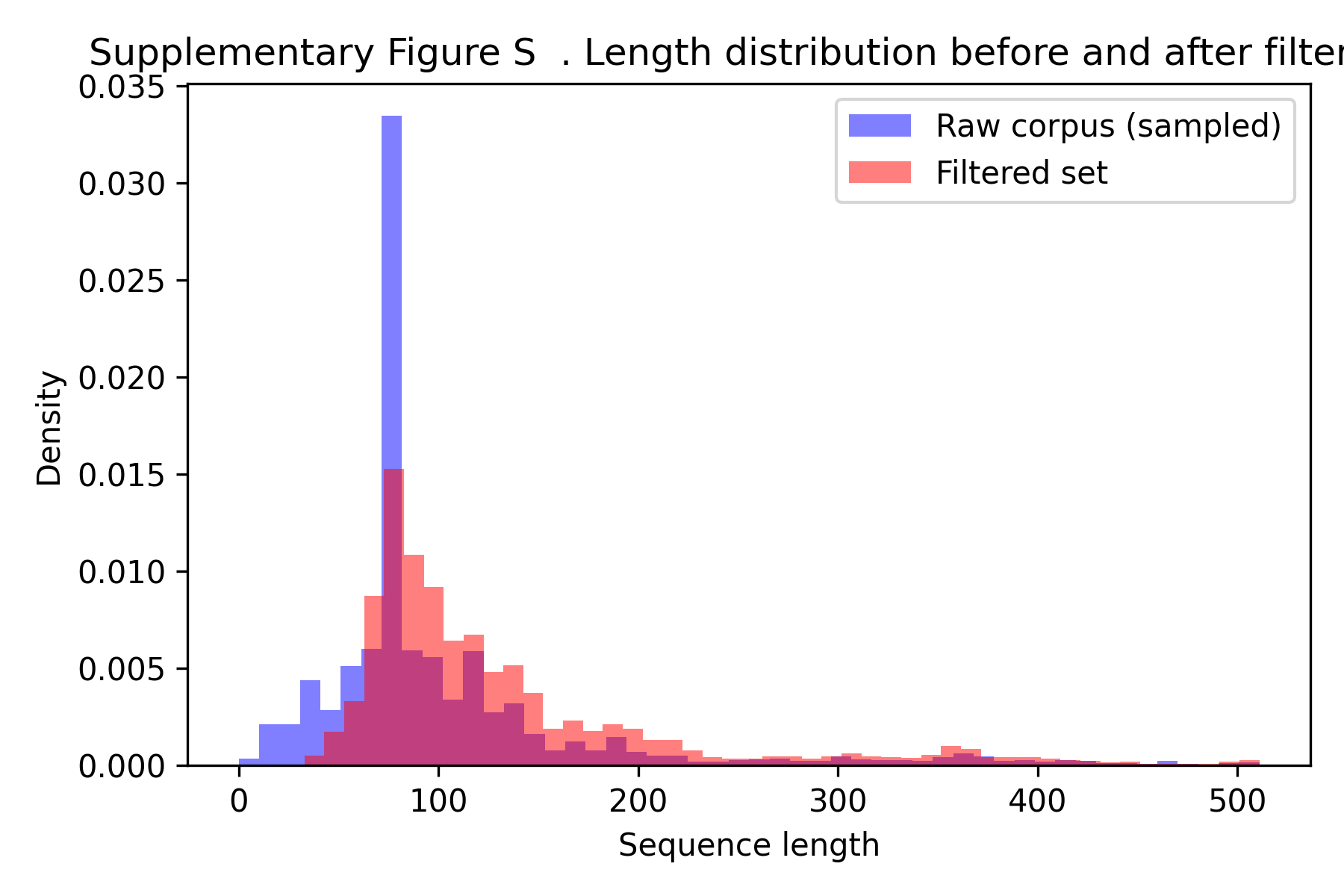

Supplement: btaf527_Supplementary_Data [file btaf527_supplementary_data.zip › Supplementary material/Supplementary Materials for Online Figures S2.bmp]

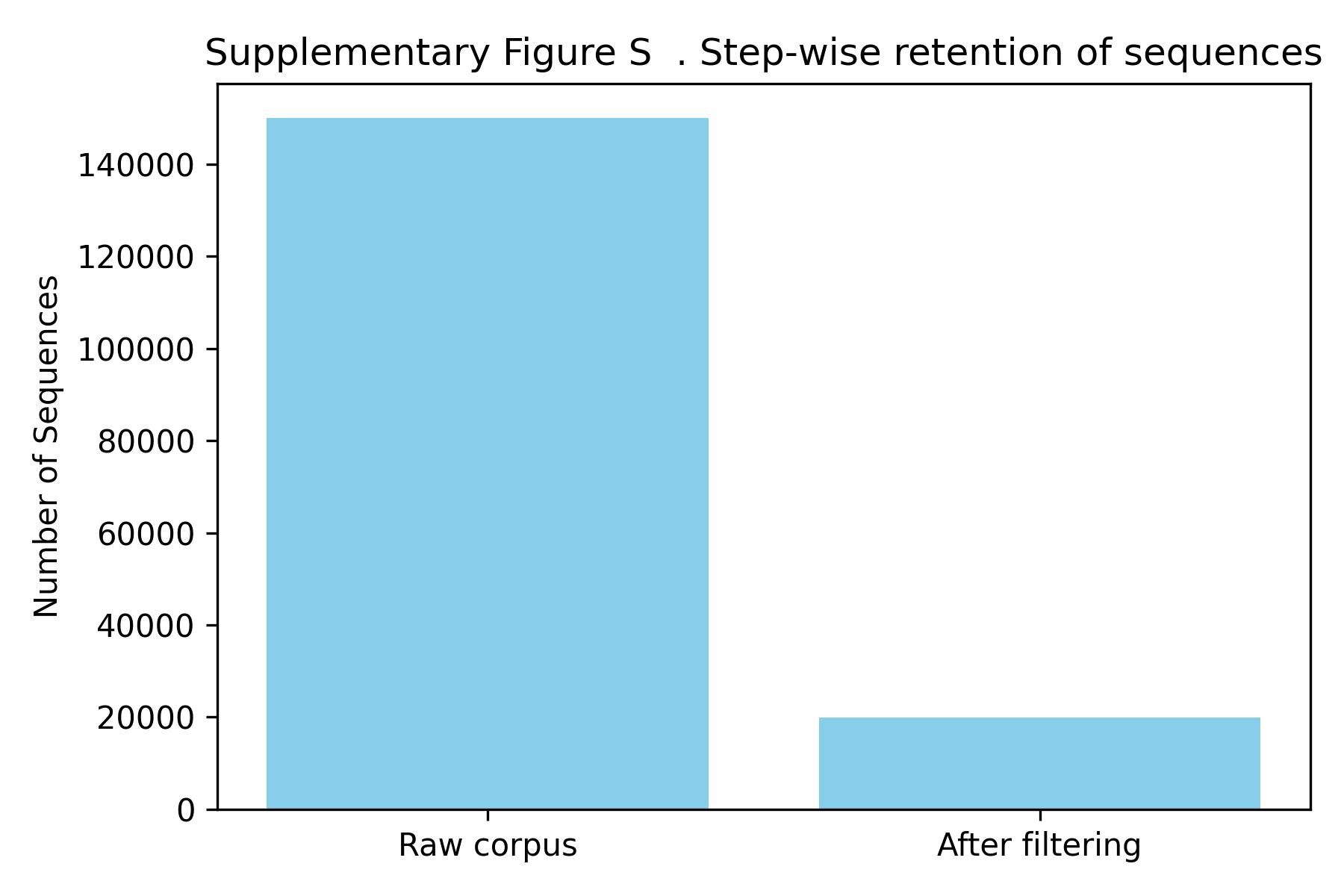

Supplement: btaf527_Supplementary_Data [file btaf527_supplementary_data.zip › Supplementary material/Supplementary Materials for Online Figures S3.bmp]

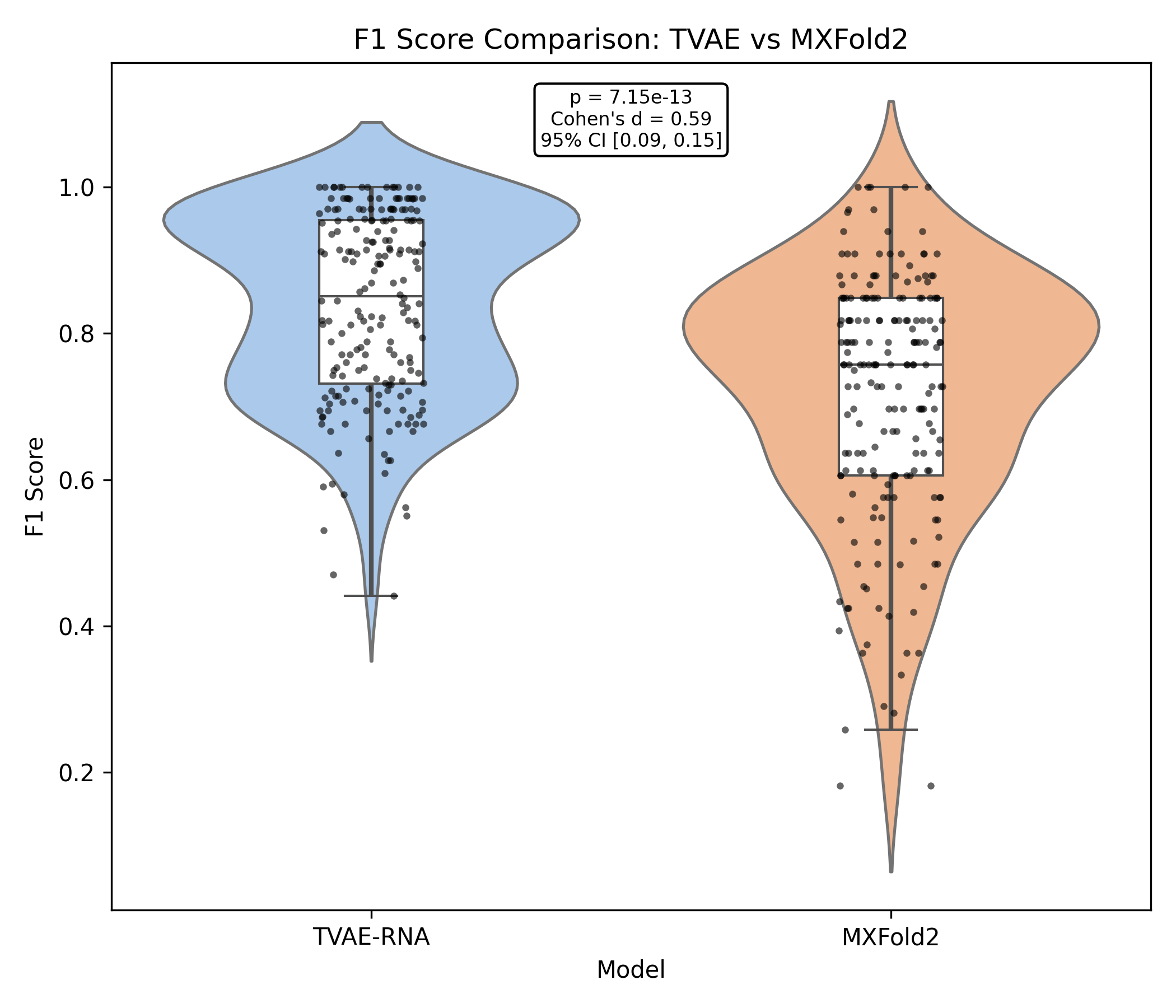

Supplement: btaf527_Supplementary_Data [file btaf527_supplementary_data.zip › Supplementary material/Supplementary Materials for Online Figures S4.png]

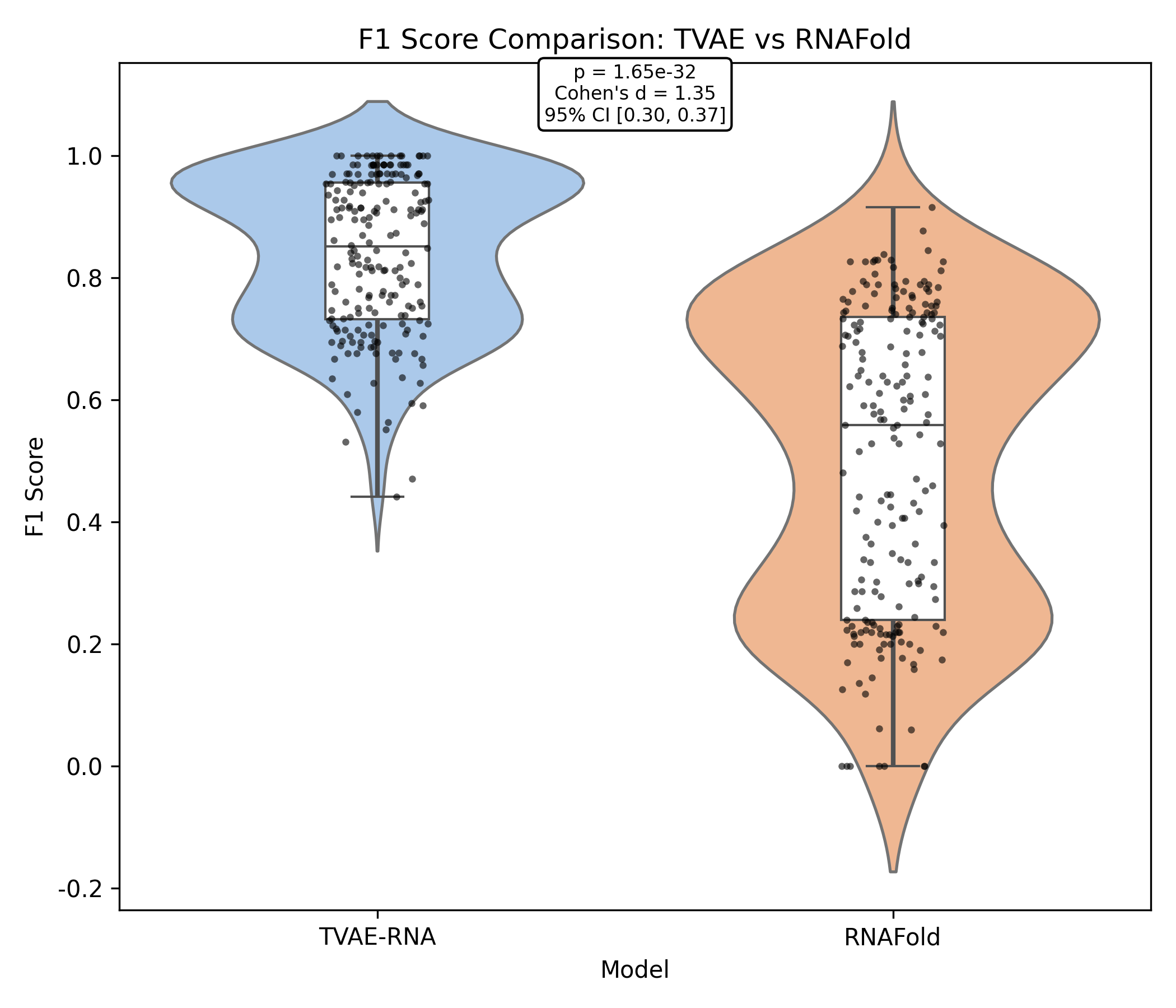

Supplement: btaf527_Supplementary_Data [file btaf527_supplementary_data.zip › Supplementary material/Supplementary Materials for Online Figures S5.png]

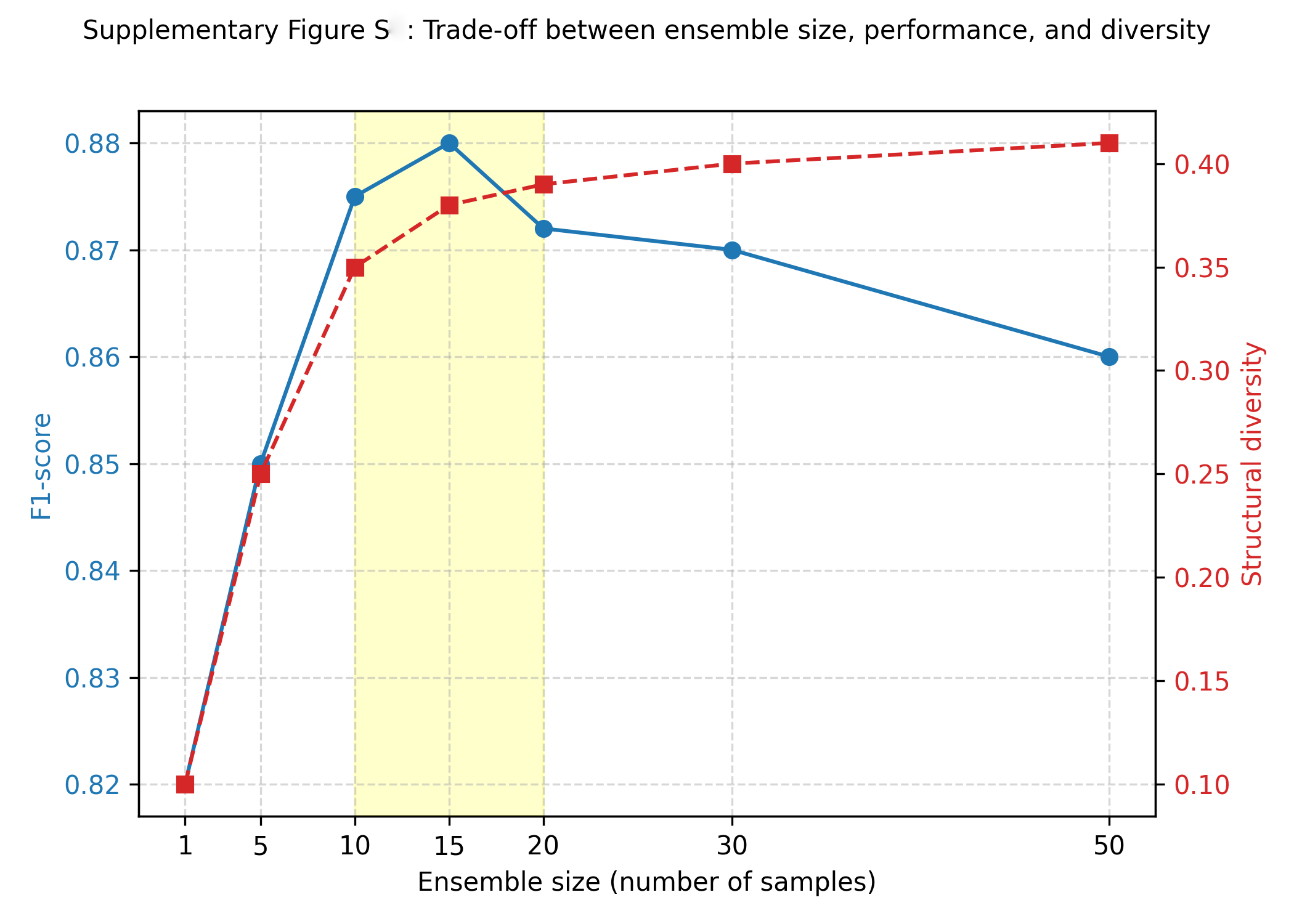

Supplement: btaf527_Supplementary_Data [file btaf527_supplementary_data.zip › Supplementary material/Supplementary Materials for Online Figures S6.bmp]
